# Lateral Traumatic Esophago-Cutaneous fistula in a Child; Platelet-Rich Fibrin Glue Challenge

**DOI:** 10.5812/ircmj.7975

**Published:** 2013-03-05

**Authors:** Marjan Joudi, Daryoush Hamidi Alamdari, Mehran Hyradfar, Hamid Reza Rahimi, Elena Saremi, Mahdi Fathi, Reza Shojaeian, George Koliakos

**Affiliations:** 1 Pediatric Surgery Department, Sheikh Academic Hospital, Mashhad University of Medical Sciences, Mashhad, IR Iran; 2 Stem Cell and Regenerative Medicine Research Group, Department of Biochemistry, Mashhad University of Medical Sciences, Mashhad, IR Iran; 3 Department of Modern Sciences and Technologies, Faculty of Medicine, Mashhad University of Medical Sciences, Mashhad, IR Iran; 4 Heart Surgery Departments, Imam Reza Academic Hospital, Mashhad University of Medical Sciences, Mashhad, IR Iran; 5 Departments of Biochemistry, Medical School, Aristotle University of Thessaloniki, Thessaloniki, Greece

**Keywords:** Fistula, Wounds and Injuries

## Abstract

**Introduction:**

The endoscopic fibrin glue or platelet-rich fibrin glue (PRFG) injection is an easy, safe and effective technique for the fistula. So far, the use of fibrin glue has been limited to selected cases.

**Case report:**

Our case is a three years old male child with a neck trauma resulting in a Esophago-Cutaneous fistula after a 3 month period of follow up we decided to use PRFG for this lesion after fine debridement of the fistula tract, and the surrounding fibrosed tissue twice with a one week interval. Our visit after two weeks showed complete recovery and normal general condition. A contrast study revealed complete disappearance of the lesion.

**Conclusions:**

In our case the PRFG completely resolved a long-standing fistula resistant to exhaustive conservative management. The treatment with PRFG has been proved to be effective in the selected cases and it seems that traumatic esophago-cutaneous fistula may be one of these selections. Application of fibrin sealant should be considered early in the management of these difficult clinical problems.

## 1. Introduction

Fibrin sealants were first reported in 1909; their usages adapted extensively in Europe for 20 years, and have received increased interest over the last decade in this country. The sealant functions by imitating the final stages of the coagulation cascade. There are many commercial preparations but most involve two solutions, fibrinogen and thrombin. These are applied to the tissue site and in the presence of calcium ions the thrombin cleaves the fibrinogen into fibrin monomers which then polymerize to produce the fibrin clot independent of the patient's own coagulation cascade. The sealant has been used to aid hemostasis or to enhance tissue sealing and has found use in a wide variety of circumstances in plastic, orthopedic, vascular and general surgery (1, 2).The endoscopic fibrin glue or platelet-rich fibrin glue (PRFG) injection is an easy, safe and effective technique for the fistula even caused by malignancies, and contributes to the quality of life of these patients (3). The constituents of the sealant are either bovine in origin or more commonly isolated from blood bank samples, however, although adverse events (allergic reactions) have rarely been reported there are no documented cases of viral transmission as a result of the use of fibrin sealant (1, 2). So far, the use of fibrin glue has been limited to the treatment of anal, recto-vaginal and enter cutaneous fistula (4).

## 2. Case Study

**Figure 1.**
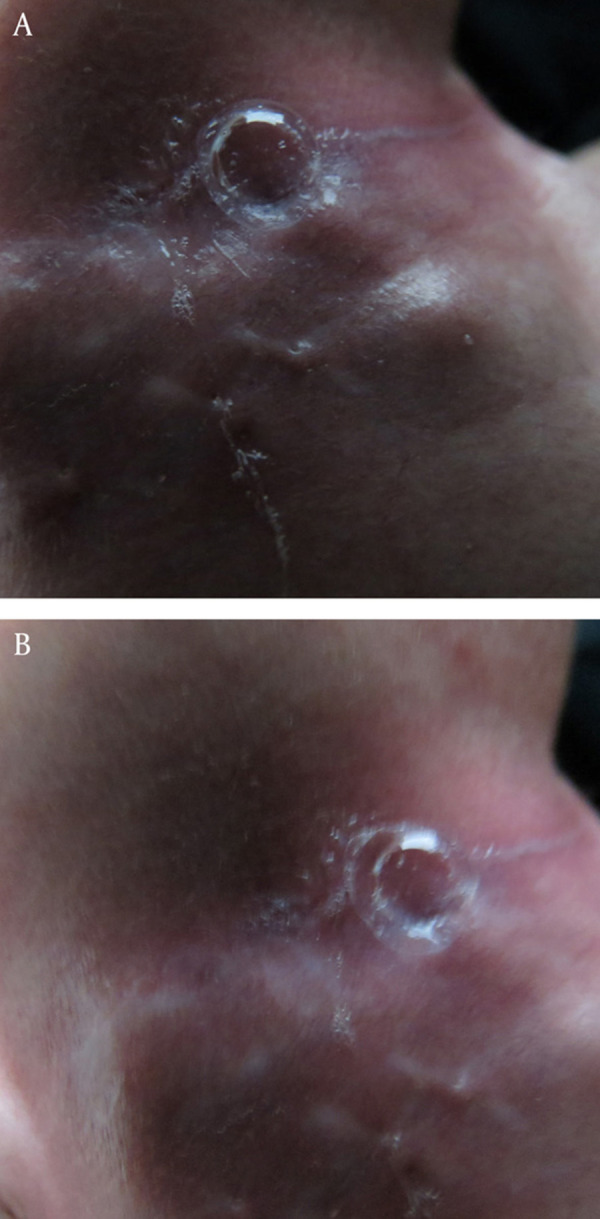
Bubble Making and Discharge for Outlet of Fistula (before Platelet-Rich Fibrin Glue injection)

**Figure 2.**
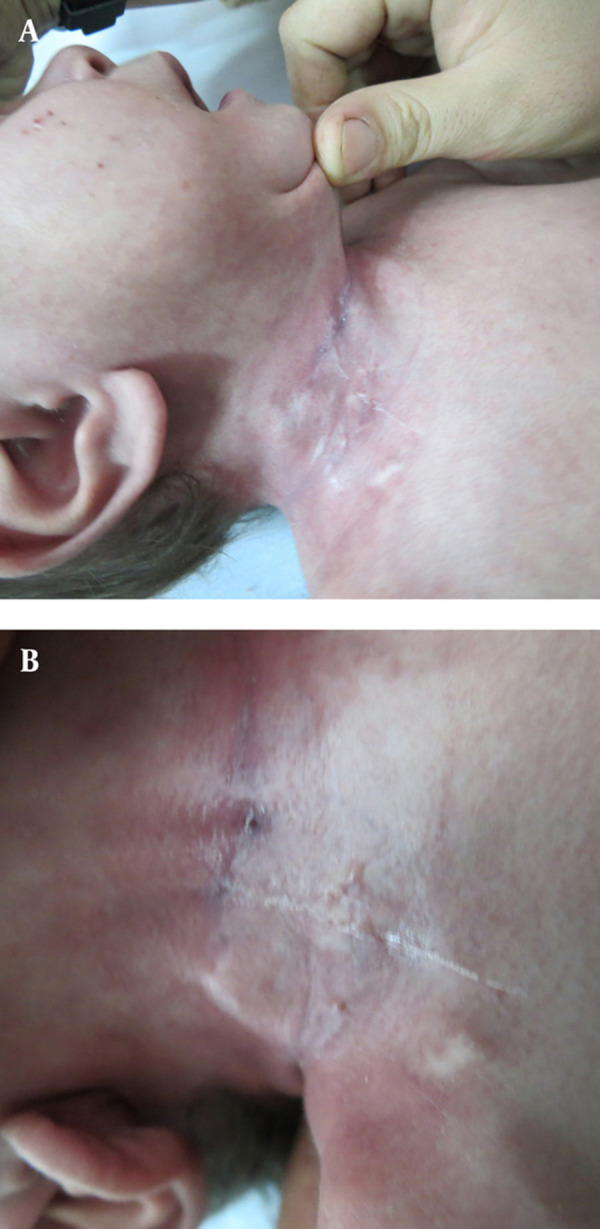
Outlet Closure by Platelet-Rich Fibrin Glue injection (after treatment)

Our case is a 3 years old male child with a neck trauma resulting in a large tissue defect in the right cervical region plus other minor injuries. Primary assessment was done, the patient was stabilized. Early surgical approach was conducted. On exploration, no major vascular or neurologic defect was found. Soft tissue repair was performed. On the third postoperative day after beginning of oral feeding, the surgeon noticed discharge from the wound. An esophago-Cutaneous fistula that wet clothes were found, the discharged fluid revealed saliva, remaining food ingestion also bubble-making (Figure 1). As the fistula was a low output one and patient’s general condition was acceptable, greatest inlet diameter was 4 mm and length of the fistula was 5 mm, other complication of this fistula was perioutlet erythema and irritation (Figure 1), Due to possible damage to the cervical vessels and nerves further surgical exploration was cancel and our primary plan was conservative management and observation. At the first step of conservative management a rigid esophagoscopy was done and in right lateral cervical esophagus inlet of fistula was seen. Output decreased but never ceased during a 3 month period of follow up. After this period we decided to use PRFG for this lesion primarily used for some other esophageal fistulas successfully by the author. A home-made PRFG was prepared apportioning was not used for avoiding probable anaphylactic shock (1) and viral inactivation also was done in process of PRFG preparation. After taking 400 ml peripheral blood from ABO match donor into commercial 450-ml triple blood donation bags and passing all viral tests safety according to blood transfusion regulation; PRFG were prepared according to standard procedures (5, 6). The platelets were prepared by first centrifugation at 2000g for 2 min and then second centrifugation at 4000g for 8 min and the supernatant plasma was separated and 10 ml platelet rich plasma was left. The fibrinogen concentrate was prepared from separated plasma by cryoprecipitating method. Following a -70 ºC freeze and a 4 ºC thaw, plasma were centrifuged at 6500 x g for 5 minutes. The supernatant plasma was removed to a final volume of 10 ml, and 15 ml concentrated fibrinogen mixed with platelets (final volume 20ml). 1 ml thrombin was prepared from removed plasma by adding 10% calcium gluconate. In each application to 5 ml mixture of fibrinogen & platelets was added 250 µl mixture of thrombin & calcium gluconate. PRFG was applied topically onto the wounds (7). So after fine debridement of the fistula tract, and the surrounding fibrosed tissue by freshening the edges of the lesion, we applied PRFG around the esophageal opening twice with a one week interval. Observation during the treatment showed that the external orifice was treated after the first procedure. Our visit after 2 weeks showed complete recovery and normal general condition. A contrast study revealed complete disappearance of the lesion (Figure 2). After 1 year follow up there are no complication or any problem with surgery site.

## 3. Discussion

This is an extremely rare condition in which a traumatic fistula has been formed connecting skin and esophagus in the lateral neck area. We searched the media using several medical search machines but we did not succeed in finding a similar case in the literature. After third operation the conservative management was disappointing, also some physicians believe that for same situation “conservative treatment should not be prolonged beyond 14 days and that endoscopic treatment should be performed at that stage with 2 - 4 ml of reconstituted fibrin glue” (8). There were cases of esophageal traumatic fistula but all of them connected the esophagus to the nearby trachea and none of them used PRFG as treatment (9-12). There is commercial fibrin glue without platelets (because of platelets elimination during fibrinogen preparation) which is used in clinic. In this treatment, home-made fibrin glue is prepared which contain platelets. Platelets, in addition their role in hemostasis, contain a number of growth factors such as platelet-derived growth factor (PDGF), transforming growth factor-β (TGF-β), vascular endothelial growth factor (VEGF), epidermal growth factor (EGF), insulin-like growth factor (IGF), and basic fibroblast growth factor (bFGF) (13-17) that stimulate cells to regenerate tissue. There are several reports that cytokines or growth factors are critical to wound healing (18). These growth factors play major roles in local inflammation, re-epithelialization, granulation tissue formation, neovascularization and extracellular matrix production from various cell sources and through diverse mechanisms (19). The child’s treatment was a great challenge but as we had the experience of using PRFG in the treatment of congenital trachea esophageal fistulas in our medical center, we decided to use it for this special case as well. In the upper gastrointestinal tract, fibrin sealant has been used in the management of acute hemorrhage, to support suture lines after resection, to aid resolution of anastomotic leaks after resection and in the treatment of esophagotracheal and gastro-colic fistulae (1, 2, 20, 21). Between 1991 and 2003 the treatment of anastomotic leaks of the upper and lower gastro-intestinal tract was performed with fibrin glue in 13 selected patients (4). Recurrent tracheo-oesophageal fistula (TEF) is the most common and serious complication of the treatment of oesophageal atresia with TEF. Following this complication, the patient might need several operations (22, 23). The mortality rate is high (22). The treatment of recurrent TEF has been done using bronchoscopic application of fibrin glue (FG) to the fistula tract (23). Fibrin glue has also been shown to be a suitable delivery vehicle for exogenous growth factors that may in the future be used to accelerate wound healing (24). Some authors suggest that if the gastro-intestinal fistulae clinically occurred 7 days after surgery a higher number of endoscopic sessions were necessary than in patients with earlier appearance of anastomotic leakage (4). We used the sealant in our case twice with a time interval of one week. Delayed treatment with endoscopic placement of fibrin glue in a persistent esophago-bronchial fistula from spontaneous rupture of the esophagus (Boerhaave's syndrome a resistant to extended conservative management was performed successfully (1). The history of failed conservative management and immediate success of the intervention leave no doubt that the fibrin treatment was responsible for the resolution of the fistula (1). In our case the fibrin glue completely resolved a long-standing fistula resistant to exhaustive conservative management. Fibrin glue has been widely used as an adhesive in plastic and reconstructive surgery (24). The treatment with PRFG has been proved to be effective in the selected cases (4) and it seems that traumatic esophago-cutaneous fistula may be one of these selections. Application of fibrin sealant should be considered early in the management of these difficult clinical problems (1).
